# Medicinal plant-based drug delivery system for inflammatory bowel disease 

**DOI:** 10.3389/fphar.2023.1158945

**Published:** 2023-03-23

**Authors:** Ningcen Li, Meijuan Wang, Zhongxi Lyu, Kai Shan, Zelin Chen, Bo Chen, Yong Chen, Xiyou Hu, Baomin Dou, Jingyu Zhang, Lifen Wang, Tianyi Zhao, Hongjiao Li

**Affiliations:** ^1^ Department of Laboratory Medicine, Nanfang Hospital, Southern Medical University, Guangzhou, China; ^2^ Research Center of Experimental Acupuncture Science, Tianjin University of Traditional Chinese Medicine, Tianjin, China; ^3^ Qingdao Hospital of Traditional Chinese Medicine (Qingdao Hiser Hospital), Qingdao, Shandong, China; ^4^ Binhai New Area Hospital of TCM, Fourth Teaching Hospital of Tianjin University of Traditional Chinese Medicine, Tianjin, China; ^5^ School of Traditional Chinese Medicine, Tianjin University of Traditional Chinese Medicine, Tianjin, China; ^6^ Institute of Basic Research in Clinical Medicine, China Academy of Chinese Medical Sciences, Beijing, China

**Keywords:** medicinal plant, traditional Chinese medicine, drug delivery, nanomedicine, extracellular vesicles, synthetic nanoparticles, plant exosome-like nanovesicles, inflammatory bowel disease

## Abstract

Inflammatory bowel disease (IBD) is a chronic recurrent intestinal disease. The incidence rate of IBD is increasing year by year, which seriously endangers human health worldwide. More and more studies have shown that medicinal plants or their main phytochemicals have great potential in the treatment of intestinal diseases. However, the disadvantages of low oral absorption rate, low biological distribution and low systemic bioavailability limit their clinical application to a certain extent. In recent years, the application of nanotechnology has made it possible to treat IBD. Nanoparticles (NPs) drug delivery system has attracted special attention in the treatment of IBD due to its small size, low immunogenicity, surface modification diversity, targeting and other advantages. Synthetic nanoparticles and extracellular vehicles (EVs) can deliver drug components to colon, and play a role in anti-inflammation, regulation of oxidative stress, improvement of intestinal flora, etc. In addition, some medicinal plants can secrete EVs by themselves, and carry biological molecules with therapeutic effects to act on the intestine. Some clinical trials to evaluate the safety, tolerance, toxicity and effectiveness of EVs-loaded drugs in IBD are also progressing steadily. This review introduces that synthetic nanoparticles and medicinal plants derived EVs can play an important role in the treatment of IBD by carrying the effective active phytochemicals of medicinal plants, and discuss the limitations of current research and future research needs, providing a scientific and reliable basis and perspective for further clinical application and promotion.

## 1 Introduction

Inflammatory bowel disease (IBD) is a chronic recurrent intestinal disease, which consists of ulcerative colitis (UC) and Crohn’s disease (CD). UC affects the colon, while CD can affect any part of the digestive tract from the mouth to the perianal area (Ochsenkuhn and D'Haens, 2011). IBD is a global disease. In recent years, the incidence rate of IBD has increased year by year, not only in Western countries, but also in developing countries in South America, Asia, Africa and Eastern Europe, which might bring social and economic burden on governments and health systems (Ng et al., 2017). It is believed that its evolution can be divided into four epidemiological stages: disease emergence, disease acceleration, disease deterioration and disease balance (Kaplan and Windsor, 2021). UC and CD share many common pathological and clinical features but their treatment methods are completely different, and their pathogenesis is still unclear (Flynn and Eisenstein, 2019). The current research shows that heredity (host susceptibility) (Mohanan et al., 2018), environment (microorganisms) (Sun et al., 2019), barrier factors (intestinal epithelial and innate immune cells) (Haag and Siegmund, 2015) and other causes may participate in the pathogenesis of IBD. Although traditional drugs used to treat IBD, such as anti-inflammatory drugs, immunosuppressants and glucocorticoids, have been proved to be effective in correcting immune dysregulation and dampening inflammation within the intestinal mucosa they still have some disturbing side effects (toxicities to healthy organs, allergic reactions, and nausea, etc.) (Zhang et al., 2017).

With the development of science and technology, more and more evidence proved that medicinal plants themselves or their main phytochemicals have great potential for the treatment of intestinal diseases. But the shortcomings of low oral absorption rate, low solubility and low bioavailability make it difficult to approve these effective ingredients as drugs in clinical practice. Therefore, it is an important way to find more accurate and effective drug delivery methods. In recent years, the application of nanotechnology has made it possible to treat IBD (Verstockt et al., 2018). Nanoparticle (NP) drug delivery systems have attracted special attention in the treatment of IBD due to their small size, low immunogenicity, stability, surface modification diversity, targeting and other advantages (Zhang et al., 2017). Since the 1990s, synthetic nanoparticles have been widely used in clinical drug delivery (Witwer and Wolfram, 2021). Besides, Extracellular vesicles (EVs)-based therapy is one of the most concerned one at present. EVs are cell-derived membranous structures comprising exosomes (30–200 nm), microvesicles (MVs) (50–1000 nm) and apoptosis bodies (50–2000 nm), which are originated from the endosomal system or released from the plasma membrane or apoptotic cells, respectively. They contain a variety of functional contents mainly proteins, nucleic acids and lipids (van Niel et al., 2018). The EVs used in the research mostly come from mammals, but the possibility of immunogenicity limits their development to a certain extent. EVs derived from natural plants may alleviate the concerns of most mammalian exosomes, while medicinal plants with therapeutic effects themselves may play a greater role in transportation (Karamanidou and Tsouknidas, 2021). The methods of loading drugs into EVs mainly include indirect loading through the source cells and direct loading of EVs after separation. The EVs secreted by varieties cells treated with medicinal plants or effective ingredients can specifically target the disease site, increase the drug content in the colon, prolong the drug retention time in the body, affect the intestinal microenvironment, and reduce the related systemic adverse reactions while improving the efficacy. The highly selective homing ability and specific targeting potential of EVs make them an ideal tool for targeted therapy of IBD. In addition, some medicinal plants with anti-inflammatory effects can secrete EVs, which called plant exosome-like nanovesicles (PELNVs) and carry biological molecules with therapeutic effects to act on intestinal cells (Dad et al., 2021). This review introduces the therapeutic efficacy of EVs and synthetic nanoparticles as a therapeutic drug-delivery loaded with medicinal plant phytochemicals or medicinal plant derived EVs for IBD to clarify its mechanism. At the same time, some ongoing clinical trials for evaluating the safety, tolerance, toxicity and effectiveness of enterovirus loaded drugs in IBD are introduced. These findings provide a scientific and reliable basis for the role of medicinal plant related EVs in IBD and contribute to further clinical application and promotion.

## 2 Application of synthetic nanoparticles loaded with medicinal plant active ingredients in IBD

Engineering nanomaterials are widely used in the treatment of IBD. Nanoparticle delivery systems (including transferosomes, liposomes, dendrimers, mesoporous silica, solid lipids, microspheres and cellular carriers, etc.) may facilitate targeted delivery of drugs, increase effective concentration and reduce the side effects (Yang and Merlin, 2019). The actions are summarized below and shown in Table 1.

**TABLE 1:** Physicochemical characterization and application of NPs as delivery nano-platforms for phytochemicals.

Refs	Drugs	Size (nm)	Nanoparticles	Diseases	Model used	Test sites	Biochemical measurements
Iglesias et al. (2019)	RES	170 (± 90) nm	Chitosan-based composites	IBD	*in vitro*	—	—
Gandhi et al. (2020)	RES	—	Chitosan-based microsponges	Acetic acid-induced colitis	*in vivo*/vitro	Colon tissues	Mucosal ulceration↓, inflammatory cell infiltration↓, submucosal edema↓, goblet hyperplasia↓
Pujara et al. (2021)	RES	165 (± 2) nm	β-Lactoglobulin-Nanosphere-Encapsulated	Spontaneous colitis	*in vivo*/vitro	Colon tissues	IL-10↑, TNF-α↓, IL-1β↓, IL-17↓
Naserifar et al. (2020)	RES	120 ± 7 nm 131 ± 9 nm	PLGA	TNBS-induced colitis	*in vivo*/vitro	Colon tissues	TNF-α↓, SOD↓, MPO↓, IL-6↓
Ohno et al. (2017)	Cur	—	Theracurmin	DSS-induced colitis	*in vivo*	Colonic mucosa, faeces	TNF-α mRNA↓, IL-1β mRNA↓, IL-6 mRNA↓, CXCL1 mRNA↓, CXCL2 mRNA↓, NF-κBp65↓
Plaza-Oliver et al. (2020)	Cur	173 ± 20 nm	NE-ADP	—	*in vitro*	Caco-2 cell	ROS↓
Salah et al. (2022)	Cur	65 nm	NPL	DSS-induced colitis	*in vivo*/vitro	Caco-2 cell, colon tissues	IL-1β↓, IL-6↓, IL-8↓, IL-10↑
Sharma et al. (2019)	Cur	210.56 ± 41.22 nm	SBLNs	DSS-induced colitis	*in vivo*/vitro	Stomach, small intestine, colon	MPO↓, TNF-α↓, protein carbonyl group↓, LPO↓
Wang et al. (2022)	Cur	200 nm	AceKGM	DSS-induced colitis	*in vivo*/vitro	Colon tissues	MPO↓
Mutalik et al. (2016)	Cur	425 nm	PAAm-g-XG copolymer	Acetic acid-induced colitis	*in vivo*/vitro	Colon tissues, plasma	MPO↓, NO↓
Beloqui et al. (2014)	Cur	166 ± 3.0 nm	PLGA/ES100 NPs	DSS-induced colitis	*in vivo*/vitro	Caco-2 cell, colon tissues	TNF-α↓, MPO↓
Diez-Echave et al. (2021)	QT	175.8 ± 0.9 nm	QSFN	DSS-induced colitis	*in vivo*/vitro	Colon tissues	TNF-α↓, IL-1β↓, IL-6↓, MCP-1↓, ICAM-1↓, NLRP3↓, iNOS↓
Khater et al. (2022)	QT	—	CS	DSS-induced colitis	*in vivo*	Colon tissues	ROS↓, NO↓, MDA↓, H_2_O_2_↓, GSH-Px↑, SOD↑, IL-6↓, TNF-α↓, IFN-γ↓, IL-10↓, CAT↑, MUC-2↑, JAM-2↑, Occludin↑, Nrf2↑, HO-1↑, CD4↓, CD8↓, TLR4↓, iNOS↓, COX2↓
Shen et al. (2021)	QT	220 nm	pH/ROS dual-responsive prodrug micelle	DSS-induced colitis	*in vivo*/vitro	Colon tissues	IL-6↓, TNF-α↓, iNOS↓
Feng et al. (2021)	RMP	205.6 ± 1.86 nm	PLGA	DSS-induced colitis	*in vivo*/vitro	Colon tissues	IFN-γ↓, IL-6↓, IL-10↑, ZO-1↑, occludin↑, acetate↑, propionate↑, butyrate↑
Feng et al. (2021)	RMP	202 nm	PLGA	LPS-induced colitis	*in vivo*/vitro	Jejunum tissues	TNF-α↓, IL-6↓, IL-1β↓, PGE2↓
Feng et al. (2022)	SK	190.3 nm	ES100/HA/CS	TNBS-induced colitis	*in vivo*/vitro	Colon tissues	TNF-α↓, IL-6↓, IL-1β↓, COX-2↓, iNOS↓, IL-10↑, TGF-β↑, ZO-1↑, occludin↑
Zhao et al. (2021)	BBR	230.2 ± 18.1 nm	CS	DSS-induced colitis	*in vivo*/vitro	Colon tissues	Firmicutes↑, proteobacteria↓, TNF-α↓, IL-6↓, TGF-β↓, IL-23↓
Zhang et al. (2018)	Shogaol	249.6 ± 1.3 nm	PLGA/PLA-PEG-FA	DSS-induced colitis	*in vivo*/vitro	Colon tissues	TNF-α↓, IL-6↓, IL-1β↓, iNOS↓, Nrf-2↑, HO-1↑
Nidhi et al. (2017)	Embelin	12.6 ± 2.1 μm	Microspheres	Acetic acid-induced colitis	*in vivo*/vitro	Colon tissues	MPO↓, MDA↓, LPO↓, GSH↑
Tambuwala et al. (2019)	PCT	210–288 nm	Albumin	DSS-induced colitis	*in vivo*/vitro	Colon tissues	p65↓, HIF-1α↓, INF-γ↓, IL-6↓, TNF-α↓, MPO↓
Nguyen et al. (2021)	SM	110 nm	siRNP	DSS-induced colitis	*in vivo*/vitro	Colon tissues	NO↓, IL-1β↓, IL-6↓, TNF-α↓, 2,2-diphenyl-1-picrylhydrazide radical↓
Varshosaz et al. (2015)	SM	109 ± 6 nm	Eudragit RL PO NPs	Acetic acid-induced colitis	*in vivo*/vitro	Colon tissues	TNF-α↓, IL-6↓, MPO↓
Miroliaee et al. (2011)	SM	245 ± 82.47 nm	Se	TNBS-induced colitis	*in vivo*	Colon tissues	TNF-α↓, IL-1β↓, NF-κB↓, MPO↓, lipid peroxidation↓, protein carbonyl↓

Notes: ↑, upregulated by drugs; ↓, downregulated by drugs. Abbreviations: PLGA, poly (lactic-co-glycolic acid); PAAm-g-XG, pH-sensitive hydrolyzed polyacrylamide-grafted-xanthan gum; NPs, nanoparticles; NE, nanoemulsions; ADP, ascorbyl-2,6-dipalmitate; OC-B, MPO, myeloperoxidase; NO, nitric oxide; NPL, crosslinked starch nanocarrier; SBLNs, solid binary lipid nanoparticles; PLGA, poly (lactide-co-glycolide) acid; MUC-2, mucin-2; JAM, junction adhesion molecule; Nrf2, nuclear factor erythroid 2-related factor 2; HO-1, hemeoxygenase-1; TLR4, toll-like receptor 4; QSFN, Quercetin-loaded silk fibroin nanoparticles; RMP, ramulus mori polysaccharide; CS, chitosan; HA, hyaluronic acid; ES100, Eudragit S100; GSH, glutathione; LPO, lipid peroxides concentration; siRNP, silica-containing redox nanoparticles; Se, Selenium; SCFA, short chain fatty acids.

### 2.1 NPs for the delivery of resveratrol

Resveratrol (RES), a natural (poly) phenol, which exists in the traditional medicinal plant called *Polygonum cuspidatum* Sieb. et Zucc. [Polygonaceae; Polygoni Cuspidati Rhizoma et Radix] (Medium Taxonomic Confidence) and has been proven to prevent and improve intestinal inflammation by interacting with NF-κB, SIRT1, mTOR and HIF-1α, etc., (Nunes et al., 2018). However, the poor water solubility, rapid metabolism and low bioavailability of RES limit its clinical applications (Gowd et al., 2022). Some studies have used chitosan-based composites materials as the carrier of RES and found that RES can be continuously released in the colon, which may have a potential therapeutic effect on IBD. Hydrogel is used as a matrix for controlled release of bioactive molecules to ensure good biocompatibility of biomaterials. Chitosan can tightly adhere to the wall surface of gastrointestinal tract (GIT), and temporarily open the tight connection between epithelial cells, enhancing the drug absorption of intestinal epithelial cells. The cross-linking in the composite can fully inhibit the 3D network of hydrogel and the water flow in the system. The chitosan network is responsible for significantly reducing the drug release rate, making this system a multifunctional tool by extending the retention and delivery time. This study carried out 20 experiments and a Box⁻Behnken experimental design was used to evaluate the importance of these independent variables related to packaging efficiency (EE). It was found that at RES/polymer ratio of 0.75:1 w/w, the RES EE value could be enhanced in 24 h and 39°C (Iglesias et al., 2019). In other studies, 3^3^ Box Behnken was further used to design colon targeting system to optimize the preparation of RES loaded chitosan-based microcapsules. It was found that 3^3^ Box Behnken could enhance the therapeutic effect of UC. Average weight, friability test (%), hardness are used as evaluation parameters of matrix tables, and hardness of 4.13 ± 0.13 kg/cm^2^, friability below 0.69% ± 0.23% and average weight of 499.65 ± 1.35 mg would be satisfactory. This study used appropriate mathematical models to fit drug release data, with the increase of drug concentration, the percentage yield (% Y) of the prepared microsponges and drug loading (DL %) have an increasing trend, which may be related to the increase in viscosity of the dispersed phase and reduced the diffusion rate from viscous solutions into aqueous phase (Gandhi et al., 2020). When RES was encapsulated in β-lactoglobulin (BLG) nanospheres, it was found that the complexation of RES with BLG increased the solubility and stability of RES. RES with BLG increased the aqueous solubility of RES by ≈ 1.7 times with 10% w/w loading. After oral administration, BLG-RES (50 mg/kg) significantly improved the body weight percentage and disease activity index (DAI) of UC mice, upregulated the expression of IL-10, increased the number of goblet cells, restored the destruction of colon epithelium, and produced anti-inflammatory effect (Pujara et al., 2021). In order to protect RES from rapid degradation and increase its intestinal permeability, researchers have developed resveratrol-loaded galactosylated poly (lactic-co-glycolic acid) (PLGA) nanoparticles targeted by folic acid (FA). The release of RES, PLGA-RES and PLGA-FA-RES were 4.5%, 61% and 99% respectively, whether the PLGA encapsulation could retain RES under simulated gastric conditions (HCl 0.1 N, pH 1.2) or release a large amount of RES under simulated intestinal conditions (PBS, pH 7.4). It was found that oral FA-PLGA- RES and PLGA- RES (100 mg RES) could significantly inhibit inflammation (downregulating TNF-α, SOD, MPO and IL-6) and reduce the accumulation of neutrophils and lymphocytes. Compared with non-targeted delivery system, FA-targeted system has the highest efficacy in inhibiting colitis (Naserifar et al., 2020).

### 2.2 NPs for the delivery of curcumin

Curcumin (Cur) is a bioactive ingredient derived from *Curcuma longa* L. [Zingiberaceae; Curcumae longaae rhizoma] (High Taxonomic Confidence). Traditionally, it has been used to flavor various delicacies in Southeast Asia and Arabian countries (Baliga et al., 2012). Cur has a variety of biological functions, such as anti-inflammatory, anti-cancer, anti-oxidation, and hypoglycemic, etc. A large number of studies have proved that Cur may be interacted with NF-κB, JAKs/STATs, MAPK, PPARγ and TRPV1 to treat IBD and slow down the progress of IBD (Karthikeyan et al., 2021). However, due to of its poor absorption in the GIT, poor stability, low bioavailability and rapid elimination, its traditional application in treatment is limited. Oral a newly developed nanoparticle curcumin (named Theracurmin) can inhibit NF-κB activation and mucosal Tregs induction (increasing expansion of CD4^+^ Foxp3^+^ regulatory T cells and CD103^+^ CD8α-regulatory dendritic cells) in colon epithelial cells, downregulate the expression of mucosal mRNA of inflammatory mediators (TNF-α mRNA, IL-1β mRNA, IL-6 mRNA, CXCL1 mRNA, CXCL2 mRNA), regulate the intestinal flora environment (increasing the abundance of butyrate-producing bacteria and fecal butyrate), further significantly alleviate weight loss, DAI, histological colitis score, and improve mucosal permeability in DSS-induced colitis mice. Although some studies have shown that the absorption efficiency of nanoparticle curcumin in rats and humans is 30-fold higher than that of curcumin powder, this study has not verified the drug loading rate, release rate and safety of nanoparticle curcumin, only compared the efficacy of nanoparticle Cur with that of pure Cur (Ohno et al., 2017). Oxidative stress reaction plays an important role in the development of IBD. In consideration of the antioxidant effects of α-tocopherol nanoemulsions (NE) (a lipophilic antioxidant to scavenge free radicals in hydrophobic environments) and ascorbyl-2,6-dipalmitate (ADP) (maintains the antioxidant properties of ascorbic acid). Some researchers combined these two materials to construct a new type of antioxidant nano lotion nano carrier. They can be combined to construct a new antioxidant nanoemulsion as a novel nanocarrier for delivering Cur in order to play a synergistic role. The study evaluated the stability of NE-ADP in temperature, storage time, normal (non-enzyme-riched) and normal simulated gastric and intestinal fluids (SGF and SIF respectively). It was found that NE-ADP could stably exist in gastrointestinal media and be located in Caco-2 cells, which may play a role in IBD by down regulating intracellular reactive oxygen species (ROS) level (Plaza-Oliver et al., 2020). Crosslinked starch nanocarrier loaded with Cur (NPL/Cur) can also be delivered to TNFα-stimulated Caco-2 epithelial cells, further inhibits the pro-inflammatory cytokines such as IL-1β, IL-6, IL-8 and increases the expression of anti-inflammatory cytokine IL-10. Oral administration of NPL/Cur can target the colon and deliver Cur deeper into the epithelium to alleviate the symptoms of DSS-induced colitis mice. This study used an *ex vivo* murine colonic explant from an inflammation model and found that NPL/Cur is not only on the surface of epithelial cells, but also can penetrate into the intestinal wall, with stronger targeting (Salah et al., 2022). Curcumin encapsulated by solid binary lipid nanoparticles (C-SBLNs) also has good gastrointestinal stability, and prolonged drug release up to 24 h. Oral administration of C-SBLNs can reduce leukocyte infiltration, oxidative stress and proinflammatory cytokines (TNF-α), restore colon structure in DSS-induced colitis mice (Sharma et al., 2019). Acetylated konjac glucomannan (AceKGM) NPs loaded with Cur were prepared by emulsion solvent evaporation technology, which has the targeting property of colon macrophages. It is selected pH1.2 to simulate SGF and pH7.4 to simulate SIF respectively. It was found that Cur-AceKGM NPs did not release significantly in SGF but released more than 60% in SIF within 24 h, suggesting that Cur-AceKGM NPs may be a suitable delivery carrier for colon targeting. At 48 h, the release of Cur-AceKGM NPs in SIF can reach 81%. Cur-AceKGM NPs can decrease the colon local level of MPO and DAI score, significantly alleviate the symptoms of colitis (Wang et al., 2022). Drug delivery systems based on pH and/or enzyme also have good application prospects in IBD. The pH-sensitive hydrolyzed polyacrylamide-grafted-xanthan gum (PAAm-g-XG) nanoparticles were used to load Cur for colon delivery, and it was found that Cur could be released optimally in the pH 6.8 solution (Compared at pH 1.2, 4.5, 6.8 and 7.2 respectively). Oral Cur/PAAm-g-XG/NPs can reduce the levels of myeloperoxidase (MPO) and nitrite, prevent weight loss, and alleviate the symptoms of acetic acid-induced IBD in rats (Mutalik et al., 2016). Using pH-sensitive polymeric NPs combining both PLGA and a polymethacrylate polymer to delivery Cur can significantly enhanced the penetration of Cur in Caco-2 cell monolayer. In the medium with pH 1.2 and 4.5, CC was not released, but once the pH value reached the neutral value, CC was released rapidly. Oral administration of Cur-NPs can significantly reduce neutrophil infiltration and TNF-α, restore colon structure in DSS induced colitis mice (Beloqui et al., 2014).

### 2.3 NPs for the delivery of quercetin

Quercetin (QT) is a strong antioxidant related to kaempferol, which is expressed in more than 100 kinds of medicinal plants, such as *Panax notoginseng* (Burkill) F.H.Chen [Araliaceae; Notoginseng radix et rhizoma] (High Taxonomic Confidence), Platycladus orientalis (L.) Franco [Cupressaceae, Platycladi cucumen] (High Taxonomic Confidence), *Ginkgo biloba* L. [Ginkgoaceae, Ginkgo folium] (High Taxonomic Confidence), etc. It can prevent oxidative damage and cell death by scavenging oxygen free radicals, inhibiting xanthine oxidase, lipid peroxidation and chelating metal ions (Boesch-Saadatmandi et al., 2011). Because QT can be absorbed in the stomach and small intestine, metabolized rapidly or degraded strongly, that may lead to less QT entering the colon and limiting its potential use for IBD treatment. The application of nano carriers may well solve this problem. Oral QT-loaded silk fibroin nanoparticles (QSFN) can produce obvious intestinal anti-inflammatory properties by down regulating proinflammatory cytokines (TNF-α, IL-1β, IL-6, MCP-1, ICAM-1, NLRP3, iNOS) and significantly reducing the DAI score of DSS-induced colitis mice. < 40% of loaded QT is released in the GST, while the rest can reach the colon, actively enhancing its anti-inflammatory effect in the damaged area (Diez-Echave et al., 2021). The incidence rate and progression of IBD are closely related to oxidative stress caused by excessive production of ROS. CS loaded with QT (QT-NPs) can downregulate inflammatory cytokines (IL-6, TNF-α, IFN-γ, IL-10) in colitis mice, upregulate tight junction protein (MUC-2, JAM-2, occludin), regulate oxidative stress state (ROS, NO, MDA, H2O2, GSH-Px, SOD, CAT, Nrf2, HO-1), restore the healthy structure of colon tissue in DSS-induced colitis mice. This study also compared the effects of three concentrations of QT-NPs (10 mg/kg, 15 mg/kg, 20 mg/kg), and found that higher concentrations had better effects, showing a concentration dependence (Khater et al., 2022). Some studies have further improved the inflammation targeting function on the basis of CS materials, such as pH/ROS dual-responsive prodrug micelle (GC-B-QT). Anti-inflammatory single drug QT was conjugated to glycol chitosan by aryl boronic ester which can give potential pH/ROS dual responsiveness. In the presence of H_2_O_2_, the release rate of GC-B-Que under physiological conditions is low (<20 wt%), but it contains 10% at pH 5.8 μM H2O2 was almost completely released in the medium (>95 wt% after 72 h). GC-B-QT micelles tend to accumulate in intestinal inflammatory sites and inhibit inflammatory cytokines (TNF-α, IL-6, iNOS) and showed better therapeutic effect than free drugs (QT and mesalazine) (Shen et al., 2021).

### 2.4 NPs for the delivery of ramulus mori polysaccharide

Because flora can affect intestinal immune homeostasis, the role of intestinal flora in IBD has been paid more and more attention. Ramulus mori polysaccharide (RMP) is one of the main components of *Morus alba* L. [Moraceae, Mori ramulus] (Medium Taxonomic Confidence). Its surface is porous and spongy and composed of seven monosaccharides: mannose, rhamnose, glucuronic acid, glucose, xylose, galactose and arabinose. It has good anti-inflammatory, antioxidant and hypoglycemic effects. It can also regulate the structure of intestinal flora and prevent intestinal inflammation damage. However, as a non-starch polysaccharide, RMP has low oral bioavailability and short biological half-life, which constructing a nano delivery system may help solve this problem (Yu et al., 2019). RMP was encapsulated into PLGA to form PLGA-RMP. It is found that oral PLGA-RMP can inhibit the expression of IFN-γ and IL-6, upregulate the expression of IL-10, ZO-1 and occludin, adjust the metabolic disorder (up regulating the content of acetate, propionate and butyrate, reducing the diversity and richness of intestinal microbiota), repair intestinal barrier and finally prevent weight loss, reduce the DAI score, and promote the recovery of colon length in DSS-induced colitis mice. This study only observed the effectiveness of PLGA-RMP on IBD and explored its mechanism, but there was no *in vitro* experiment to evaluate the concentration, drug loading rate and release rate of PLGA-RMP (Feng et al., 2021). In mice with IBD induced by LPS, oral administration of PLGA-RMP can also regulate macrophage polarization, inhibit specific inflammatory cytokines (including TNF-α, IL-6, IL-1β, PGE2), inhibit the activation of CD3^+^CD8^+^ T cells, increase the number of activated Treg in the intestine, and reduce the DAI score and intestinal inflammatory damage. In order to test the potential toxicity of PLGA-RMP, this study treated RAW264.7 macrophages with different concentrations of PLGA-RMP. It is found that PLGA-RMP was non-toxic to macrophages at 125 μg/mL and RMP encapsulated by PLGA show a higher uptake efficiency by macrophages than free RMP (Feng et al., 2021).

### 2.5 NPs for the delivery of shikonin

Shikonin (SK) was originally extracted from *Arnebia euchroma* (Royle ex Benth.) I.M.Johnst. [Boraginaceae, Arnebiae radix] (Medium Taxonomic Confidence), which has the functions of inhibiting inflammation, regulating immunity and healing wounds. Studies have shown that SK can inhibit NF-κB and STAT-3 pathway to attenuate acute UC in Balb/C mice induced by DSS (Andujar et al., 2012). It can also promote intestinal wound healing *in vitro via* induction of TGF-β release in intestinal epithelial cells (Andujar et al., 2013). SK loaded ES100/HA/CS nanoparticles (SK@SAC) were constructed using CS, hyaluronic acid (HA) and pH responsive polymer Eudragit S100 (ES100), and drug loading efficiency was 6.6%. In TNBS induced IBD mice, oral administration of SK@SAC can reduce the non-specific distribution of other organs by increasing the local drug concentration in the colon. SK@SAC has significant therapeutic effect, that can downregulate proinflammatory mediators (TNF-α, IL-6, IL-1β, COX-2 and iNOS), upregulate anti-inflammatory cytokines (IL-10, TGF-β) and tight junction proteins such as ZO-1 and occludin (Feng et al., 2022).

### 2.6 NPs for the delivery of berberine

Berberine (BBR) is a quaternary ammonium alkaloid isolated from the medicinal plant *Coptis chinensis* Franch. [Ranunculaceae, Coptidis rhizoma] (Medium Taxonomic Confidence), which is the main effective component against bacteria. It is also expressed in *Phellodendron chinense* C.K.Schneid. [Rutaceae, Phellodendri chinensis cortex] (Medium Taxonomic Confidence), etc. Modern research found that it also has analgesic, anti-inflammatory, anti-cancer, hypoglycemic, anti-hyperlipidemia and other pharmacological effects, which is widely used in IBD. However, the oral absorption of BBR is poor. After injection, BBR quickly enters various organs and tissues. The blood concentration is maintained soon, and the blood concentration is lower than the minimum inhibitory concentration (Habtemariam, 2016). The nanoparticle was constructed stablely from a phenylboronic esters-modified carboxylmethyl chitosan (OC-B) conjugated with BBR (OC-B-BBR) which could respond to the selective degradation of ROS. In the slightly excessive ROS environment, the stimuli-responsive borate ester of OC-B-BBR can be broken to release BBR molecules, and more than 90% of BBR can be released in 24 h incubation. OC-B-BBR can suppress the secretion of some inflammatory cytokines such as TNF-α, IL-6, TGF-β, IL-23 and remodel intestinal microbiota in DSS-induced mice, significantly improved the symptoms and colon injury (Zhao et al., 2021).

### 2.7 NPs for the delivery of shogaol

Shogaol is the major pharmacologically active compounds of ginger which is broadly used for a wide range illness worldwide. Ginger is the rhizome of *Panax ginseng* C.A.Mey. [Araliaceae, Ginseng radix et rhizoma] (High Taxonomic Confidence), a well-known plant with anti-inflammatory and antioxidant properties. Ginger can inhibit various pro-inflammatory cytokines and the inflammation related pathways, such as TLRs, NF-κB, STATs, NLRPs, MAPKs, and mTOR, which may play an effective anti-inflammatory role in IBD (Lashgari et al., 2022). The active compound 6-shogaol was loaded on PLGA/PLA-PEG-FA nanoparticles (NPs-PEG-FA/6-shogaol). It was found that NPs-PEG-FA/6-shogaol could be absorbed by colon-26 cells and activated Raw 264.7 macrophages. PEG modification can improve the biocompatibility of NPs. NPs, NPs-PEG and NPs-PEG-FA will not change colon-26 cells (24 h–48 h) at any test concentration (up to 1 mg/mL), but NPs can reduce the cell viability of Raw 264.7 macrophage cells at 48 h, while there is no decrease in NPs-PEG or NPs-PEG-FA treatment groups. In addition, NPs-PEG-FA seems to have no obvious toxicity *in vivo*, and has no obvious effect on gastrointestinal tract and main organs such as heart, liver, spleen, lung and kidney. It was found that NPs-PEG-FA/6-shogaol can be taken up by colon-26 cells and activated Raw 264.7 macrophages. Oral NPs-PEG-FA/6-shogaol can downregulate the proinflammatory factors (TNF-α, IL-6, IL-1β and iNOS), upregulate the expression level of anti-inflammatory factors (Nrf-2 and HO-1), alleviate the colitis symptoms and accelerate the wound repair of DSS-induced colitis mice (Zhang et al., 2018).

### 2.8 NPs for the delivery of embelin

Embelin is a kind of benzoquinone compound extracted from *Embelia ribes* Burm.f.[Primulaceae, Emblica ribes] (Medium Taxonomic Confidence). It is distributed in the east of India to Indonesia and China, which has been used to treat fever, inflammatory diseases and various gastrointestinal diseases for a long time. Modern research has found that embelin also has anti-tumor, anti-inflammatory and analgesic properties. Embelin can significantly reduce the DAI score, inflammatory factors, MPO accumulation and further alleviate weight loss, diarrhea, massive hemorrhage and immune cell infiltration in DSS-induced colitis mice (Kumar et al., 2011). The targeting and effectiveness of embelin can be significantly improved by delivering it *via* nanoparticle loading. Embelin-loaded enteric-coated microspheres can significantly reduce the ulcer activity score, oxidative stress level (downregulate the expression of MPO, MDA, LPO), upregulate the level of glutathione, and reduce the inflammatory reaction in acetic acid-induced colitis rats. Compared with other conventional dosage forms, this method can produce time-dependent and pH-dependent continuous embelin release. Moreover, due to the existence of pH-sensitive and time-controllable polymers, it can effectively prevent embelin release into the gastric environment (pH 1.2) (Nidhi et al., 2017).

### 2.9 NPs for the delivery of piceatannol

Piceatannol (PCT) is a resveratrol analogue, mainly derived from the medicinal plants such as Rheum palmatum L. [Polygonaceae, Rhei radix et rhizoma] (Medium Taxonomic Confidence) and *Cinnamomum cassia* Presl [Lauraceae, Cinnamomi ramulus] (Medium Taxonomic Confidence). It is found that PCT has a good therapeutic effect on intestinal inflammation by regulating the activity of transcription factors such as NF-κB, Nrf2, HIF-1α (Ashikawa et al., 2002). PCT also has anti-coliform effect. However, due to the extensive phase II liver metabolism (glucuronization and sulfation) *in vivo*, the bioavailability of PCT is very low. Therefore, improving the bioavailability of PCT is a key concern. Some researchers have used colon-targeted capsule (colon-targeted PCT) to significantly improve the efficacy of PCT (Yum et al., 2015). Compared with capsules, nanoparticles show more prominent advantages, which can provide better healing and convenience, decrease toxicity profiles and reduce cost (Jin et al., 2018). PCT and caffeic acid phenethyl ester (CAPE, from honey bee propolis) with anti-inflammatory effect were loaded onto albumin nanoparticles, and it was found that PCT/CAPE-loaded albumin NPs could enhance the anti-inflammatory potential. PCT content has certain influence on particle size, polydispersity index and zeta potential, but the increase of CAPE content has no significant effect on particle size and polydispersity index. It can downregulate INF-γ, IL-6, TNF-α, MPO level, further alleviate weight loss, improve DAI and colon morphology in DSS-induced colitis (Tambuwala et al., 2019).

### 2.10 NPs for the delivery of silymarin

Silymarin (SM), a natural flavonoid lignan compound, is an active substance extracted from the dried fruit of the medicinal plant *Silybum marianum* (L.) Gaertn. [Asteraceae, Silybi fructus] (Medium Taxonomic Confidence). Its main components are silybin, isosilybin, silidianin and silychristin. Modern research has found that SM has the functions of antioxidation, liver protection, anti-tumor, and anti-cardiovascular/cerebrovascular diseases. However, due to poor oral bioavailability, the applicability of SM has not been recognized (Al-Drees and Khalil, 2016). SM loaded silica-containing redox nanoparticles (siRNP) can not only improve the bioavailability and colon targeting in colon mucosa of SM, but also enhance the anti-inflammatory and antioxidant capacity. SM@siRNP can scavenge 2,2-diphenyl-1-picrylhydrazide radical, inhibit NO and proinflammatory cytokine (IL-1β, IL-6, TNF-α), significantly improved the damage of colonic mucosa in DSS-induced colitis mice (Nguyen et al., 2021). The optimised nanoparticles had a loading efficiency of 98.3% ± 12% and release efficiency of 40.8% ± 5.5% at 24 h. Eudragit RL PO nanoparticles loaded with SM (75 mg/kg/day) can significantly reduce TNF-α, IL-6 and MPO activity in colon tissues, improve macroscopic and histopathological scores of acetic acid-induced colitis mice (Varshosaz et al., 2015). Selenium (Se) nanoparticles have more bioavailability with less toxicity. Combined administration of SM and nano Se can improve anti-inflammatory and antioxidant capacity by reducing the expression of TNF-α, IL-1β, NF-κB, MPO, lipid peroxidation and protein carbonyl, finally relieve symptoms of TNBS-induced colitis. Combined therapy may be a good way to treat IBD. However, more basic and clinical studies are needed to confirm the safety and effectiveness of this new combination (Miroliaee et al., 2011).

## 3 Application of medicinal plant derived EVs in IBD

Although liposomes and other nanoparticles have been used to deliver a variety of therapeutic drugs, but due to their synthetic characteristics, they still have the possibility of time-consuming, low solubility, particle aggregation and toxicity. The optimal design of nanoparticles delivery systems may require a level of complexity similar to the biological environment in order to successfully pass barriers, including clearance, degradation and physical barriers. The type, orientation, density and surface modification of ligands are all factors affecting the successful delivery of nanoparticles. EVs with unique homology and low immunogenicity have better biocompatibility and bioavailability than the traditional synthetic nanoparticles prepared in the chemical environment. At present, most of the EVs used as therapeutic carriers are derived from mammalian cells, but there are relatively few studies on the use of these EVs to load medicinal plants active ingredients for IBD. Modern research has found that not only mammals, but also plants can secrete EVs, which can be obtained with low cost and efficiency without carrying human pathogens. PELNs may have better application prospects in future research, especially the EVs derived from medicinal plants. For example, exosome-like nanovesicles with high yield (17.5 mg/kg) can be isolated from ginger (Witwer and Wolfram, 2021; Li et al., 2022). The actions are summarized below and shown in Table 2.

**TABLE 2:** Extraction, physicochemical characterization and application of EVs derived from medicinal plants.

Refs	Medicinal plants	Isolation	Size (nm)	Morphology	Diseases	Model used	Test sites	Biochemical measurements
Zhang et al. (2016)	Ginger	DGUC	231.6–292.5 nm	Cup-shaped	DSS-induced colitis	*in vivo*/vitro	Colon tissues	TNF-α↓, IL-6↓, IL-1β↓, IL-10↑, IL-22↑, E-cadherin↑, CAR1↑
Yin et al. (2022)	Ginger	DGUC	156 ± 36 nm	Spherical	LPS-induced colitis	*in vivo*/vitro	Caco-2 Cells	TNF-α↓, IL-6↓, IL-8↓, NF-κB↓
Mao et al. (2021)	Ginger	DGUC	284.6 nm	Spherical	DSS-induced colitis	*in vivo*/vitro	Colon tissues	TNF-α↓, IL-6↓, IL-1b↓, IL-10↑, IL-22↑, NLRP3↓, caspase-1↓, IL-1β↓
Zhang et al. (2017)	Ginger	DGUC	232.7 nm	Cup-shaped	DSS-induced colitis	*in vivo*/vitro	Colon tissues	CD98↓
Liu et al. (2022)	Turmeric	DGUC	178 nm	Cup-shaped	DSS-induced colitis	*in vivo*/vitro	Colon tissue	TNF-α↓, IL-6↓, IL-1β↓, NF-κB↓, HO-1↑
Gao et al. (2022)	Turmeric	DGUC	191.7 ± 15.8 nm, 243.9 ± 13.9 nm, 800.5 ± 66.2 nm	Spherical	DSS-induced colitis	*in vivo*/vitro	Colon tissue	TNF-α↓, IL-6↓, MCP-1↓
Zu et al. (2021)	Tea leaves	DGUC	140.0 nm	Spherical	DSS-induced colitis	*in vivo*/vitro	Colon tissue	ROS↓, TNF-α↓, IL-6↓, IL-12↓, IL-10↑

Notes: ↑, upregulated by drugs; ↓, downregulated by drugs. Abbreviations: DGUC, Differential density gradient centrifugation; CAR1, carbonic anhydrase 1; HO-1, hemeoxygenase-1; ROS, reactive oxygen species; MCP-1, onocyte Chemoattractant Protein 1

### 3.1 Ginger derived EVs

The EVs from ginger, which called ginger exosome-like nanoparticles (GELNs), have the property of targeting intestinal tract. In DSS-induced colitis, oral GELNs can target intestinal macrophages and intestinal stem cells (ISC), increase the survival and proliferation of intestinal epithelial cells, reduce the expression of proinflammatory cytokines (TNF-α, IL-6, IL-1β), and increase the expression of anti-inflammatory cytokines (IL-10, IL-22) and E-cadherin, thus restoring intestinal barrier dysfunction and homeostasis. Furthermore, *in vitro* and *in vivo* wound-healing models, it is demonstrated that GELNs can promote intestinal mucosa healing, upregulate the expression of carbonic anhydrase 1 (CAR1) on the surface of colon intestinal epithelial cells, reestablished normal levels of pro-/anti-inflammatory cytokines and MPO activity, and restored IEC proliferation-apoptosis balance in the intestinal mucosa (Zhang et al., 2016; Zhang et al., 2016). Similarly, for LPS-induced intestinal inflammation, GELNs can be specifically absorbed by intestinal cells through the role of caveolin, further downregulate NF-κB, IL-6, IL-8 and TNF-α in colon tissues, alleviate inflammation (Yin et al., 2022). Some study has compared the EVs derived from four plants such as grape, grapefruit, ginger, carrot, and found that only the macrophages treated with GELNs can significantly increase the expression of HO-1 and IL-10 at the same time, promote Nrf2 nuclear translocation, and also appropriately stimulate the expression of pro-inflammatory factor IL-6 to maintain homeostasis (Mu et al., 2014). Taking advantage of the good compatibility of PELNVs, some drugs were loaded into GELNs to target or synergize the treatment of IBD. Loading TNF-α with GELNs can downregulate NLRP3, caspase-1, IL-1β, TNF-α, IL-6 and IL-1b, upregulate anti-inflammatory substances such as IL-10 and IL-22 in colon tissue of DSS-induced colitis mice. This method avoids adverse reactions caused by immunosuppression in routine administration. In this study, mesoporous silicon, a type of inorganic framework with good biocompatibility, used as a suitable nanocore to embed into GELNs for solving the shortcomings of low drug loading and poor stability (Mao et al., 2021). siRNA antibody CD98 (siRNA-CD98) was loaded into GELNs to construct siRNA-CD98/GELNs. In mice with UC, oral administration of siRNA-CD98/GELNs can effectively target colon tissue, leading to the decrease of effective expression of CD98 in colon, so as to play a role in treating UC (Zhang et al., 2017; Sung et al., 2019; Sung et al., 2020).

### 3.2 Turmeric derived EVs

Using EVs as delivery carriers can make curcumin more stable, with higher concentration in the blood, higher availability of biological tissues, and stronger anti-inflammatory effect (Sun et al., 2010; Aqil et al., 2017). In acute inflammation induced by LPS, turmeric-derived nanoparticles (TDNPs) showed excellent anti-inflammatory and antioxidant properties. It is proved that oral TDNPs can reduce proinflammatory cytokines (TNF-α, IL-6 and IL-1β), upregulate antioxidant gene HO-1 to relieve colitis in mice and accelerate colitis regression. Further this study was conducted through NF-κB-RE-Luc transgenic mice to demonstrate that inactivation of the NF-κB pathway may the mechanism of TDNPs-treated colitis (Liu et al., 2022). Oral TDNPs can also show excellent anti-inflammatory effects in DSS-induced UC mice by restoring the damaged intestinal barrier, regulating the intestinal microbiota and polarization of and macrophages (promoting the transformation of M1 to M2 phenotype) (Gao et al., 2022).

### 3.3 Tea leaf derived EVs

Tea originated in China and was first used as a sacrifice. Later, it was used in daily diet, beverages and medical treatment in the world. Tea contains polyphenols, flavones, catechin, caffeine, inositol, folic acid, pantothenic acid and other healthy ingredients. Studies have proved that tea polyphenols can effectively eliminate free radicals in the body, with anti-aging, anti-radiation, anti-cancer, antibacterial and bactericidal effects (Khan and Mukhtar, 2018). Green tea polyphenols (GTPs) can block the transcription of NF-κB activation and upstream of mediated I kappa B kinase complex pathway activities, inhibit the invasion of cytokines and the synthesis of cyclooxygenase-2 (COX-2), further downregulate endotoxin-mediated TNF-α production to treat IBD and its complications (Rahman et al., 2018). Oral administration of the EVs derived from tea leaves can reduce the production of ROS, inhibit the expression of proinflammatory cytokines (TNF-α, IL-6, IL-12), increase the anti-inflammatory activity of macrophages (IL-10), inhibit the inflammatory intestinal response, restore the damaged colon barrier, enhance the diversity and overall abundance of intestinal microbiota, and thus prevent or alleviate IBD and colitis related colon cancer. At the same time, it was found that the galactose groups on EVs surface could be specifically internalized by macrophages through galactose receptor mediated endocytosis (Zu et al., 2021).

## 4 Discussion

IBD is a chronic inflammatory disease involving the small intestine and colon. Common symptoms include abdominal pain, diarrhea, fever, vomiting, bloody stool and weight loss, and there is a risk of colorectal cancer (CRC). Because the lesion is mainly located in the colon, colon targeted drug delivery system (DDS) has received extensive attention in the treatment of IBD. Oral administration has become the most preferred method for the treatment of IBD due to its high safety, high compliance and low cost-effectiveness. Conventional preparations, such as capsules, tablets and solutions, are used clinically to deliver anti-inflammatory drugs or immunosuppressants for the treatment of IBD (Yang and Merlin, 2019). In addition, biological agents such as TNF-α antibodies are also used to treat IBD through intravenous (IV) or subcutaneous (SC) injection. However, these methods cannot produce colon targeted drug delivery, and often cause serious systemic side effects (Li et al., 2022). Phytochemicals from medicinal plants can act on various pathogenic and inflammatory targets, but these natural molecules may damage their therapeutic activity before reaching the inflammatory colon (Khare et al., 2020). Recently, drugs-based on NPs have received extensive attention due to their potential to solve such problems. Polymer nanoparticles, such as synthetic polymer-, natural polymer-, polymeric prodrug, hybrid polymer- (including pH-responsive, ROS-responsive, dual-responsive, polymer-lipid) based NPs have been extensively studied in the treatment of IBD. Some targeted ligands, such as monosaccharides, polysaccharides, peptides, folic acid, antibodies and their fragments, can also be used to modify NPs to improve their targeting (Zu et al., 2021). The active ingredients of these medicinal plants can be better absorbed by the whole body in the form of nanoparticles or EVs. Some studies have found that the absorption rate of Cur loaded nanoparticles can be increased by about 3 times compared with pure Cur (Mutalik et al., 2016). Because patients with IBD lack physiological lipids and some lipids have immunomodulatory properties, it is hypothesized that the combination of lipids and hytochemicals in nanocarriers may be a valuable strategy to overcome IBD. Three kinds of lipid-based nanocarriers containing Cur (including self-nanoemulsifying drug delivery systems-SNEDDS, nanostructured lipid carriers-NLC and lipid core-shell protamine nanocapsules-NC) were studied and compared as anti-inflammatory drugs in DSS-induced colitis mice. The efficiency of permeability across Caco-2 cell monolayers was NC > SNEDDS > NLC and Cur suspension. At the same time, it is proved that lipid nanocarriers show increased Cur retention in the intestinal tract, rather than increased Cur permeability, which may provide some new ideas for drug delivery in the future (Beloqui et al., 2016). The synergistic use of some drugs combined with targeting and therapeutic effects has increased the efficacy and has also been applied in IBD. For example, the combination of targeted drug delivery with anti-inflammatory drugs and ROS scavengers has good advantages. Micelles formed by CUR conjugated hydroxyethyl starch as vehicles are used as carriers to further load dexamethasone (DEX) to form HES-CUR nanoparticles (DHC-NPs). DHC-NPs can target the inflammatory colon. When the α-amylase is overexpressed in the inflammatory colon can lead to HES degradation, and the drug is released by amylase reaction from DHC-NPs. Targeting and combination therapy significantly reduce the damage caused by DSS induced UC (Xu et al., 2022).

In addition to synthetic NPs, the presence of EVs also provides a direct drug delivery route to colon mucosa for the treatment of IBD. Compared with the synthetic NPs, which will lead to cell stress, apoptosis, inflammatory body activation and other side effects, EVs are non-toxic, low immunogenicity and can be produced in large scale, may have better application prospects (Hu and Palic, 2020; Radmanesh et al., 2021). In a variety of EVs, plants derived EVs contain bioactive lipids, proteins, ribonucleic acids and other pharmacologically active molecules, which can be used as natural nano carriers. Medicinal plants derived EVs not only have therapeutic effects themselves, but also can package different therapeutic drugs to produce synergistic effects (Di Gioia et al., 2020). However, due to the environmental reasons of the GIT, such as gastric acid, digestive enzymes and intestinal microbiota, the passage of EVs is threatened. Researchers have extracted natural EVs from three kinds of edible tea and found that they have high potential for large-scale production and can accumulate in colitis tissues for up to 48 h, improving the bioavailability of their contents. The particle sizes, polydispersity indices (PDIs), and zeta potentials of all the EVs presented only slight variations in simulated stomach acid and intestinal fluid. How to enhance the stability while ensuring the targeting of EVs is one of the problems to be solved at present (Zu et al., 2021). Since proteins, nucleic acid and lipids are important components of EVs, further quantitative and comparative analysis of these components may improve the understanding of medicinal plants derived EVs biogenesis and intercellular communication mediated by effective components in future research (Kim et al., 2020).

These natural nanoplatforms have great potential for medical transformation. At present, there are also clinical trials for the treatment of intestinal diseases being funded. Some researchers found that the bioavailability of Cur taken orally by IBD patients is limited even at a very high dose of 8–12 g per day. It is further proposed to solve the problem of Cur delivery by using plant exosomes to deliver drugs to colon tumors and normal colon tissues. There are also clinical trials to evaluate the effect of plant derived exosomes or effective active phytochemicals on patients with IBD (https://clinicaltrials.gov/). In a prospective randomized study, the main focus was to compare the improvement of symptoms of IBD patients with ginger exosomes alone, curcumin, and ginger exosomes plus curcumin (NO. NCT04879810). This trial included 90 patients with chronic IBD, using three main stratified factors: race (white and black), sex and IBD type (CD and UC), and using the incidence of bloody stool to justify the sample size. Although limited medicinal plant-based nano preparations have been tested in humans, but the number is few and the evidence is low, there is still an urgent need for new clinical trials to analyze non-toxic natural NPs targeting the colon in order to deliver natural products.

During the treatment of IBD, there are still some problems to be considered. First, compared with other drug delivery routes, oral drug delivery route has obvious advantages, such as systemic side effects, simple self-medication, good patient compliance, and has better research and application prospects. In the design of nano-delivery materials, there are still some aspects to be considered. Oral nano-pharmaceuticals should overcome a variety of physical, chemical and biological barriers in GIT, including high acidic environment in the stomach, extreme pH changes and proteolytic enzymes along GIT. For NPs with weak acid or base groups, it should be considered that the change of pH value will affect the changes of ionizable groups and morphology of nanoparticles. For NPs targeting the lamina propria, how to overcome the intestinal epithelium and its mucus secretion layer is a key obstacle to be considered. Due to the short residence time of NPs in the small intestine (3–4 h), this poses additional challenges for the design of this part of NPs. In addition, due to the needs of the lesion site, such as the leakage of epithelial inflammation and the loss of mucous layer, the oral nanodrugs used for the treatment of IBD may first reach the destroyed intestinal barrier and lamina propria passively. It is found that the intestinal flora disorder also plays an important role in the occurrence and development of IBD, while most symbiotic microorganisms inhabit in the mucosal layer of the intestinal cavity. Therefore, when designing some oral NPs which aiming at regulating the intestinal microbiome, targeting mucus layer should be mainly considered (Blanco et al., 2015; Zu et al., 2021). Moreover, the appropriate time for the release of drugs contained in NPs is also critical. NPs should release their drug at the target site of the intestine. Once the drug is released prematurely, it may cause side effects mediated by systemic absorption. In order to prevent premature release, it is necessary to stabilize the drug loading in the nano preparation, but in this way, the drug release will decrease with the increase of the stability of the preparation, which is also to be considered in the future. Next, although most literatures in this review have evaluated the degree of substitution, particle size, surface charge, encapsulation efficiency, loading capacity, release rate and stability of nanomaterials, and screened the best parameters. However, the targeting rate, safety and side effects of oral drugs need further exploration. Some studies only evaluated the possibility of using nanomaterials in the gastrointestinal tract and speculated that the possibility of using nanomaterials in IBD without verification *in vivo* experiments, which is also one of the shortcomings of current research. In addition, recent studies have shown that as a new way of intercellular communication, the components of EVs (including nucleic acid, protein and some other components) participate in the occurrence, development and treatment of IBD. Using EVs-like structure to design new drug formulations may provide new insights for the treatment of IBD. Because of its good biological distribution and inherent biocompatibility, it is of great interest to use EVs containing biological/chemical components as drug delivery carriers. However, at present, there are relatively few studies on EVs carrying medicinal plants and EVs derived from medicinal plants, and more animal and clinical studies are needed to study the effect of EVs on IBD. In addition, in some animal and clinical studies, it is found that combination therapy may be a good way to treat IBD. However, clinical research is needed to confirm the safety and effectiveness of this new combination.

## 5 Conclusion

**FIGURE 1:**
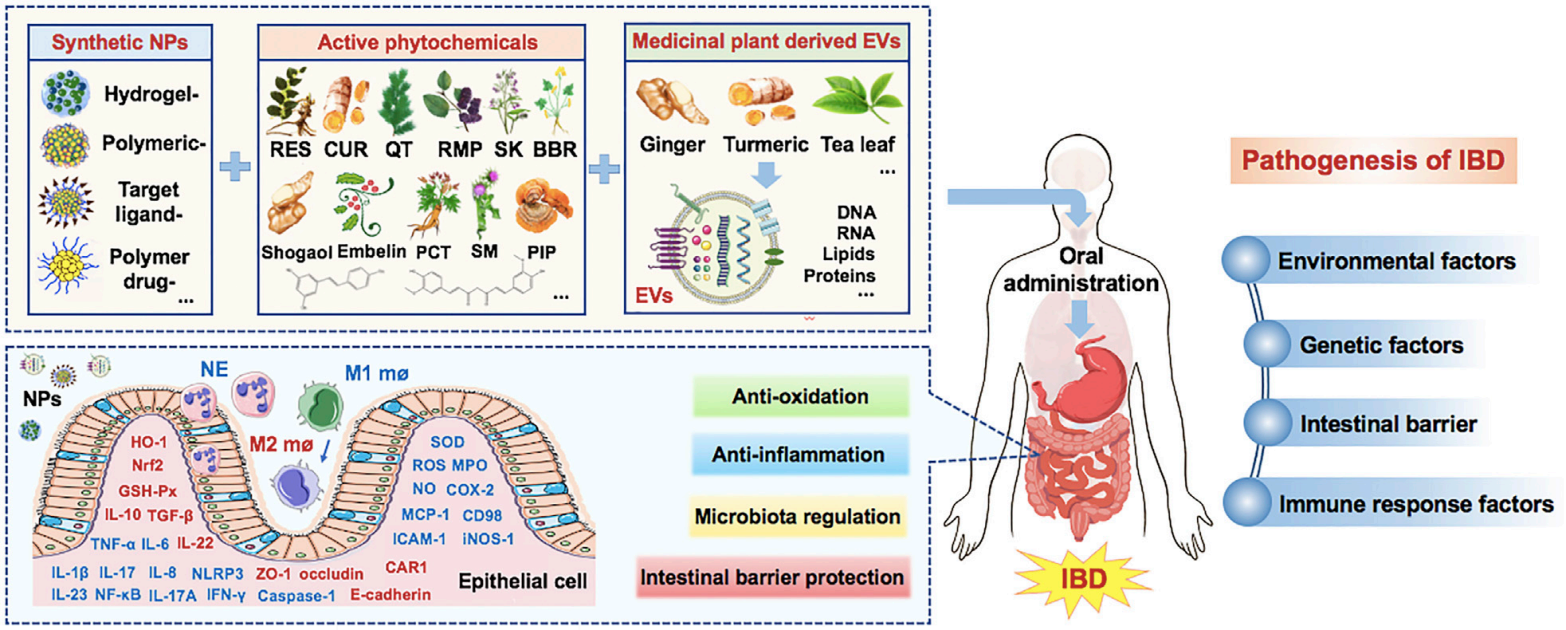
Medicinal plant-based drug delivery system for IBD. (Note: Factors in red are upregulated by NPs, while factors in blue are downregulated by NPs. IBD, Inflammatory bowel disease; EVs, extracellular vesicles; RES, resveratrol; Cur, curcumin; QT, quercetin; RMP, ramulus mori polysaccharide; SK, shikonin; BBR, berberine; ROS, reactive oxygen species; NPs, nanoparticles; CAR1, carbonic anhydrase 1; MPO, myeloperoxidase; PCT, piceatannol; SM, silymarin; Se, selenium; SOD, superoxide dismutase; MCP-1, monocyte Chemoattractant Protein 1; ICAM-1, intercellular cell adhesion molecule-1; HO-1, heme oxygenase; Nrf2, Nuclear factor erythroid 2-related factor 2).

This review demonstrates that, as an alternate, Synthetic nanoparticles and medicinal plants derived EVs can play an important role in the treatment of IBD by carrying the effective active phytochemicals. They can treat IBD by inhibiting inflammation and oxidative stress, regulating the structure of intestinal flora, and repairing the intestinal barrier. However, before they become a mature delivery system for clinical use, there are still many challenges, including how to increase drug concentration without increasing toxic and side effects, improve targeting, improve oral availability, etc. Some clinical trials to evaluate the safety, tolerance, toxicity and effectiveness of EVs-loaded drugs in IBD are also progressing steadily. Nanotechnology can play an important role in improving the therapeutic potential of phytochemicals and medicinal plant derived EVs, so as to develop new therapeutic options for IBD (Figure 1).
